# Impact of Using Different Levels of Threshold-Based Artefact Correction on the Quantification of Heart Rate Variability in Three Independent Human Cohorts

**DOI:** 10.3390/jcm9020325

**Published:** 2020-01-23

**Authors:** Juan M. A. Alcantara, Abel Plaza-Florido, Francisco J. Amaro-Gahete, Francisco M. Acosta, Jairo H. Migueles, Pablo Molina-Garcia, Jerzy Sacha, Guillermo Sanchez-Delgado, Borja Martinez-Tellez

**Affiliations:** 1 PROFITH “PROmoting FITness and Health Through Physical Activity” Research Group, Sport and Health University Research Institute (iMUDS), Department of Physical and Sports Education, Faculty of Sport Sciences, University of Granada, 18011 Granada, Spain; abeladrian@ugr.es (A.P.-F.); amarof@ugr.es (F.J.A.-G.); acostaf@ugr.es (F.M.A.); jairohm@ugr.es (J.H.M.); pablomolinag5@ugr.es (P.M.-G.); gsanchezdelgado@ugr.es (G.S.-D.); 2 EFFECTS-262 Research Group, Department of Physiology, School of Medicine, University of Granada, 18071 Granada, Spain; 3 Department of Rehabilitation Sciences, KU Leuven, University of Leuven, 3000 Leuven, Belgium; 4 Faculty of Physical Education and Physiotherapy, Opole University of Technology, 45-758 Opole, Poland; sacha@op.pl; 5 Department of Cardiology, University Hospital in Opole, University of Opole, 45-401 Opole, Poland; 6 Pennington Biomedical Research Center, Baton Rouge, LA 70808, USA; 7 Department of Medicine, division of Endocrinology, and Einthoven Laboratory for Experimental Vascular Medicine, Leiden University Medical Center, 2333 Leiden, The Netherlands

**Keywords:** Kubios software, autonomic nervous system, data processing, children, young adults, middle-aged adults

## Abstract

Heart rate variability (HRV) is a non-invasive indicator of autonomic nervous system function. HRV recordings show artefacts due to technical and/or biological issues. The Kubios software is one of the most used software to process HRV recordings, offering different levels of threshold-based artefact correction (i.e., Kubios filters). The aim of the study was to analyze the impact of different Kubios filters on the quantification of HRV derived parameters from short-term recordings in three independent human cohorts. A total of 312 participants were included: 107 children with overweight/obesity (10.0 ± 1.1 years, 58% men), 132 young adults (22.2 ± 2.2 years, 33% men) and 73 middle-aged adults (53.6 ± 5.2 years, 48% men). HRV was assessed using a heart rate monitor during 10–15 min, and the Kubios software was used for HRV data processing using all the Kubios filters available (i.e., 6). Repeated-measures analysis of variance indicated significant differences in HRV derived parameters in the time-domain (all *p* < 0.001) across the Kubios filters in all cohorts, moreover similar results were observed in the frequency-domain. When comparing two extreme Kubios filters, these statistical differences could be clinically relevant, e.g. more than 10 ms in the standard deviation of all normal R-R intervals (SDNN). In conclusion, the results of the present study suggest that the application of different Kubios filters had a significant impact on HRV derived parameters obtained from short-term recordings in both time and frequency-domains.

## 1. Introduction

Heart rate variability (HRV) refers to the variation of the time interval between R peaks (i.e., heartbeats) registered in an electrocardiogram (ECG) [1]. Previous studies have shown that a low HRV at resting conditions reflects a low modulation of the parasympathetic branch on the sino-auricular node [1,2], which is considered an indicator of cardiovascular and mortality risk [1,2,3,4]. Furthermore, it has been previously reported that HRV decreases with ageing [5,6], especially in middle-aged adults regardless of gender [6]. Beyond ECG, HRV can be estimated with certain heart rate monitors, which makes the HRV assessment more feasible (i.e., less intrusive and economically affordable).

HRV recordings using heart rate monitors are affected by technical (e.g., wrong placement of the heart rate monitor’s band) and/or biological (e.g., ectopic beats) artefacts. These artefacts could contaminate the HRV recordings, making it difficult to obtain HRV derived parameters from long-term (≈24 h) and especially short-term (≈5 min) recordings [1,7,8]. Artefacts could modify and produce important over- or under-estimation of HRV derived parameters (up to 50%) [9]. Therefore, correction of artefacts before the HRV assessment is needed for the accurate determination of HRV parameters derived from short-term recordings.

Different commercially available software have been developed to process HRV raw data [10]. The Kubios software [11,12] is one of the most-frequently used in both clinical and research settings. It allows the use of different threshold-based artefact correction filters (henceforth “Kubios filters”) [11]. The Kubios filter selection is usually based on subjective decisions since there is no consensus on what is the most appropriate Kubios filter to process HRV raw data. To our knowledge, there is only one study investigating the impact of different Kubios filters on the quantification of HRV derived parameters from short-term recordings [13]. In this study, Aranda et al. showed that the selection of the most restrictive filter had a noticeably impact on the quantification of HRV derived parameters compared with the less restrictive filter in high-level professional athletes [13]. Since HRV depends on a wide range of biological characteristics (e.g., age or body composition) [14], there is a need of determining the impact of using different Kubios filters on HRV derived parameters estimation in different populations [13]. Thus, this study aimed to analyze the impact of different Kubios filters on the quantification of HRV derived parameters from short-term recordings in sedentary children with overweight/obesity, sedentary young and sedentary middle-aged adults.

## 2. Experimental Section

### 2.1. Participants

The present study included baseline data from the ActiveBrains [15], the ACTIBATE [16] and the FIT-AGEING [17] studies. A total of 312 participants were included in this cross-sectional study: 107 sedentary children (ActiveBrains) with overweight/obesity [15], 132 sedentary young adults (ACTIBATE) [16] and, 73 sedentary middle-aged adults (FIT-AGEING) [17]. Detailed information about the methodology, inclusion and exclusion criteria of the aforementioned studies can be found elsewhere [15,16,17]. Briefly, the inclusion criteria were: (1) being physically inactive; (2) having a stable body weight; (3) not being enrolled in a weight loss program; (4) not being a smokers; and (5) not being pregnant.

All studies were conducted according to the Committee for Research Involving Human Subjects at the University of Granada (References #848 and #924 for the ActiveBrains and ACTIBATE studies, respectively), *Servicio Andaluz de Salud* (*Centro de Granada*, CEI-Granada for the ACTIBATE study) and the Human Research Ethics Committee of the *Junta de Andalucia* (0838-N-2017) for the FIT-AGEING study. All of them were performed in accordance with the Declaration of Helsinki (revision of 2013) and registered in a specific clinical trial database (i.e., Clinicaltrial.gov. IDs: NCT02295072, NCT02365129 and NCT03334357 for the ActiveBrains, ACTIBATE and FIT-AGEING study, respectively). Both, oral and written informed consent were obtained from all the participants and the children parents before their enrollment. Although the participants were enrolled in the aforementioned main studies, all of them agreed to transfer their scientific data for other scientific purposes or research studies.

### 2.2. Heart Rate Variability Assessment

**Figure 1:**
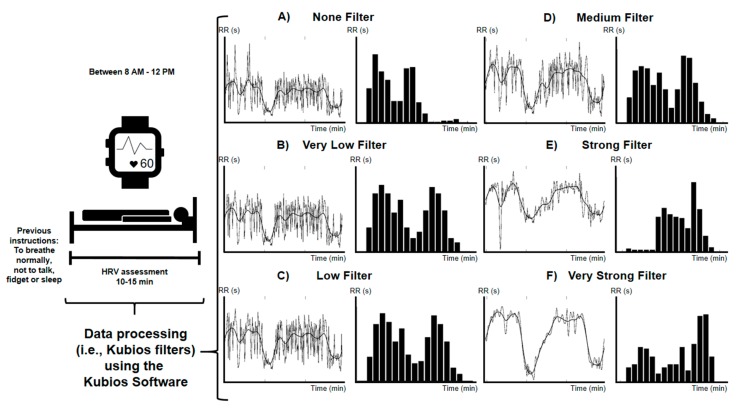
Study design. HRV: heart rate variability. None (**A**), Very Low (**B**), Low (**C**), Medium (**D**), Strong (**E**) and Very Strong (**F**) filters refers to the level of threshold-based artefact correction (i.e., Kubios filter). Graphs are examples of the same best 5 min period (of the whole heart rhythm recoding) that met the selection criteria after using different Kubios filters. RR: R-R intervals; S: seconds; min: minutes.

Participants came in the morning to the research centre by car or by bus, avoiding any physical activity before the HRV assessment. Measurements took place between 8 AM and 12 PM for the ActiveBrains study, and between 8 and 9 AM for both, the ACTIBATE and the FIT-AGEING studies. Once in the research centre, participants lay on a bed or a stretcher in supine position equipped with a Polar RS800CX heart rate monitor (Polar Electro Oy Inc., Kempele, Finland) in a quiet room with dim lighting (for all the studies), and controlled ambient temperature and humidity (for the ACTIBATE and the FIT-AGEING studies). Heart rhythm was recorded during 10–15 min (sampling frequency of 1000 Hz). Before such a recording, participants were instructed to breathe normally, and not to talk, fidget, or sleep while measurements were being taken (Figure 1). Of note, the Polar RS800CX heart rate monitor has been validated against electrocardiography, and also has been proved as a reliable equipment in the assessment of HRV in both children [18,19] and adults [20,21].

### 2.3. Heart Rate Variability Data Processing

For the HRV data processing we used the Kubios software (v.3.0.0, HRV analysis, University of Eastern Finland) [11,12]. The best 5 min period of the whole heart rhythm recording was manually selected by one evaluator based on the following criteria: (1) Gaussians R-R intervals and heart rate distribution graphs; (2) no large R-R interval outliers; and (3) R-R intervals equidistance [22,23,24]. The R-R intervals series were detrended using the smoothness prior method with alpha set at 500.

**Figure 2:**
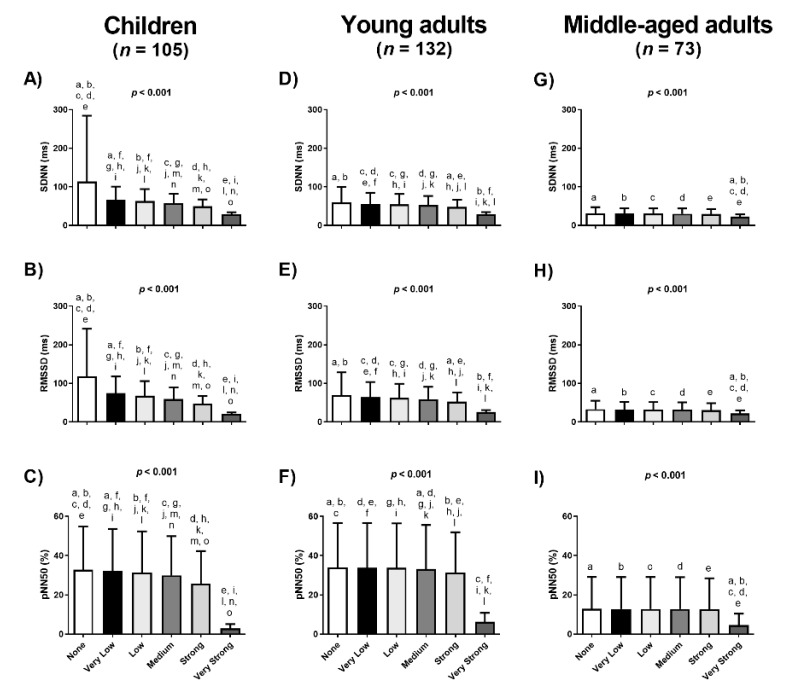
Differences on the Heart Rate Variability (HRV) time-domain parameters using different Kubios filters in three different cohorts. Data are represented as mean and standard deviation. SDNN: standard deviation of all normal R–R intervals (Panels **A, D** and **G**); RMSSD: squared root of the mean of the sum of the squares of successive normal R–R interval differences (Panels **B, E** and **H**); pNN50: number of pairs of adjacent normal R–R intervals differing by more than 50ms in the entire recording (Panels **C, F** and **I**); *p* value from the ANOVA comparisons; similar letters means Bonferroni *post-hoc* differences.

**Figure 3:**
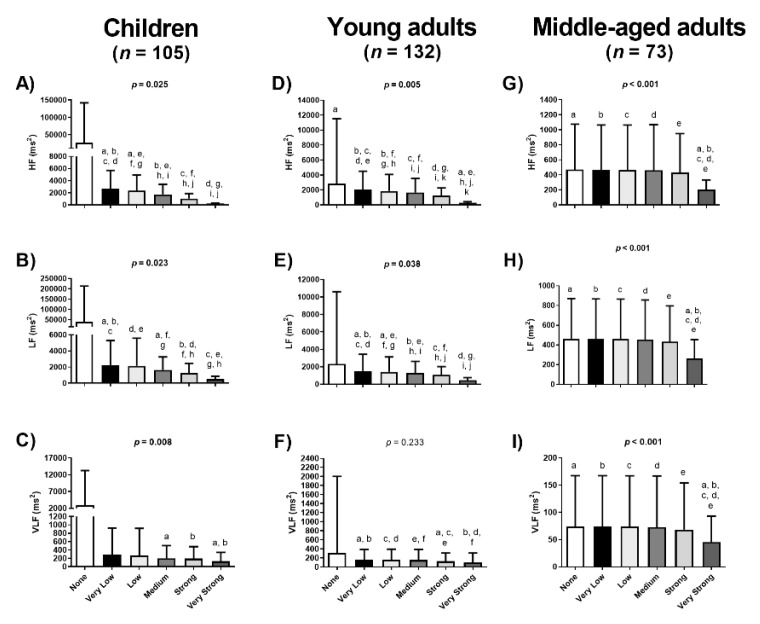
Differences on the Heart Rate Variability (HRV) frequency-domain parameters using different Kubios filter in three different cohorts. Data are represented as mean and standard deviation. HF: power in the high frequency (in absolute units, ms^2^; Panels **A, E** and **I**); LF: power in the low frequency (in absolute units, ms^2^; Panels **B, F** and **J**); LF/HF: ratio of the power in the low frequency divided by the power in the high frequency (Panels **C, G** and **K**); VLF: power in the very low frequency (in absolute units, ms^2^; Panels **D, H** and **L**). *p* value from the ANOVA comparisons; similar letters means Bonferroni *post-hoc* differences.

All Kubios filter levels (i.e., threshold-based artefact correction; Figure 1) were used on the selected 5-min periods [25]. The Kubios filter algorithm compares every R-R interval value against a local average interval [25]. The local average interval is procured by median filtering the R-R interval time series, and therefore, the local average is not influenced by outliers R-R intervals. Thus, if the R-R interval varies from the local average interval more than a specified threshold value, the interval is designated as an artefact and marked for correction by the software [25]. The available Kubios filters are: (1) None (no correction is performed; Figure 1A), (2) Very Low (0.45 s; Figure 1B), (3) Low (0.35; Figure 1C), (4) Medium (0.25 s; Figure 1D), (5) Strong (0.15 s; Figure 1E) and, (6) Very Strong (0.05 s; Figure 1F) [25]. For example, if a Very Low Kubios filter is used, the software marks for correction all R-R intervals that are 0.45 s larger or smaller than the local average interval. Then, these artefacts are subsequently interpolated using a cubic spline interpolation.

In the present study, we considered the most-frequently used HRV derived parameters from short-term recordings in both time and frequency domains. In the time-domain, we computed (1) the standard deviation of all normal R-R intervals (SDNN) in milliseconds (ms), (2) the squared root of the mean of the sum of the squares of successive normal R–R interval differences (RMSSD) in ms, and (3) the number of pairs of adjacent normal R-R intervals differing by more than 50 ms in the entire recording (pNN50) expressed as percentage. Regarding the frequency-domain, we derived (1) the power in the high frequency (HF: 0.15–0.4 Hertz (Hz)), (2) the power in the low frequency (LF: 0.04–0.15 Hz), and (3) the power in the very low frequency (VLF: 0–0.04 Hz) using the fast Fourier transformation algorithm (FFT), all expressed in absolute units (ms^2^). The LF/HF ratio was also calculated.

### 2.4. Anthropometric Assessment 

We measured the participants’ height and body weight without shoes and with light clothing using a Seca scale (model 799, Electronic Column Scale, Hamburg, Germany) and a stadiometer. Body mass index (BMI) was calculated as body weight (kg) divided by square height (m^2^). Children were classified as children with overweight/obesity accordingly to the World Obesity Federation sex-and-age specific international BMI standards [26]. Waist circumference was measured twice, in a standing position, and using a plastic tape at the midpoint between the costal margin and iliac crest in the mid-axillary line [27]. The average of both measurements was used.

### 2.5. Statistical Analysis

Descriptive data are presented as means ± standard deviation or frequency and percentages as appropriate. Normality of the HRV derived parameters in time- and frequency-domains were tested using Kolmogorov-Smirnov test and visual inspection of histograms. Although HRV derived parameters did not exhibit a normal distribution, for analytical purposes we did not transform them.

Repeated-measure analyses of variance (ANOVA) were used to test differences in HRV derived parameters in time- and frequency-domains across the different Kubios filters (i.e., None, Very Low, Low, Medium, Strong and Very Strong). Analyses were replicated with the non-parametric Friedman test and similar results were found, and therefore, only ANOVA results are reported. Bonferroni corrections were used for *post-hoc* comparisons. Analyses were conducted using the Statistical Package for Social Sciences (SPSS, v. 22.0, IBM SPSS Statistics, IBM Corporation, Chicago, IL, USA). The significance level was set at 0.05.

## 3. Results

The descriptive characteristics of the participants are presented in Table 1. The percentages of R-R intervals interpolated during the whole recording and during the selected best 5 min period for data analysis are shown in Table 2. The mean of interpolated R-R intervals across studies were 0.3, 0.7, 1.6, 4.9 and 29.9% when the Very Low, Low, Medium, Strong and Very Strong filters were used respectively (Table 2). Moreover, we performed a visual inspection of the R-R signals (Figure S1) for detecting possible premature contractions (characterized by short-long R-R intervals, i.e. coupling interval and compensatory pause) and we did not observe premature contractions in the most of the R-R signals based on the observer criteria.

Figure 2 shows mean and standard deviation values of SDNN, RMSSD and pNN50 for every cohort across the different Kubios filters. Significant differences were observed in mean SDNN (Figure 2A,D,G), mean RMSSD (Figure 2B,E,H) and mean pNN50 (Figure 2C,F,I) across Kubios filters in all cohorts (all *p* < 0.001). Significant differences were observed after *post-hoc* Bonferroni corrections in both children and young adults (Figure 2A–F). Mean SDNN, mean RMSSD and mean pNN50 were similar across Kubios filters in middle-aged adults except when the Very Strong filter was compared with the other Kubios filters (Figure 2G,H,I).

Figure 3 shows mean and standard deviation values of frequency-domain HRV derived parameters across different Kubios filters, showing similar results to those in time-domain parameters. Significant differences were observed in mean HF (all *p* < 0.025; Figure 3A,E,I) and mean LF (all *p* < 0.038; Figure 3B,F,J) across filters in the three cohorts. Significant differences were also found in the LF/HF ratio across different Kubios filters in children and young adults (all *p* < 0.001; Figure 3C,G), whereas no differences were noted in middle aged adults (*p* = 0.794; Figure 3K). Likewise, significant differences were observed in the VLF in children and in middle-aged adults (all *p* < 0.008; Figure 3D,L). Although repeated-measures ANOVA showed no differences in VLF across different Kubios filters in young adults (*p* = 0.233; Figure 3H), significant *post-hoc* differences were observed (Figure 3).

We repeated the ANOVA model to test differences in HRV derived parameters in time- and frequency-domains excluding the Very Strong Kubios filter, and the results remained similar (Figure S2 and Figure S3). Moreover, in deeper analyses we observed that the Very Strong filter equalizes HRV among the study participants although the differences between the cohorts still remained significant (Figure S4, Figure S5 and Figure S6).

## 4. Discussion

The present study showed that the application of certain Kubios filters had a significant impact on the quantification of HRV derived parameters obtained from short-term recordings in time and frequency-domains in sedentary children with overweight/obesity, sedentary young and sedentary middle-aged adults. Our results also suggest that children and young adults’ recordings were more affected by lower intensity Kubios filters (i.e., Very Low, Low and Medium) than middle-aged adults. Thus, potential artefacts could be corrected or interpolated with Kubios filters of lower intensity in children and in young adults. The Very Strong filter should be used with caution, given that the interpolated R-R intervals were 39% (from 1% to 77.2%) in children, 33% (from 0 to 75.5%) in young adults, and 17% (from 0 to 60.8%) in middle-aged adults in the selected best 5 min period, which means that most of the selected period is “artificial” (i.e., interpolated) in younger populations. Furthermore, after the visual inspection of the R-R signals in children, young adults and middle-aged adults we did not visually find any premature contractions based on the observer criteria. However, we do not have ECG tracings to verify whether “real” artefacts happened or not. Therefore, we cannot exclude that the high number of interpolated R-R intervals using the Very Strong filter could be related to either the Very Strong filter has some problem of excessive sensitivity or the heart rate monitor used for HRV assessment creates an excess of artefacts that are detected only by the Very Strong filter. Nevertheless, the former option is very likely since children and young adults usually present high sinus rhythm fluctuations resulting in big differences between consecutive R-R intervals which may be “overfiltered” by the Kubios filters. This is even more possible because the visual inspection before HRV analysis did not detect artefacts or premature contractions in the most of the R-R interval signals visually inspected.

Several studies have reported the importance of artefact correction on the quantification of HRV derived parameters from short-term recordings [8,28,29]. However, there is scarce evidence regarding the impact of using different Kubios filters on the quantification of HRV derived parameters from short-term recordings in time- and frequency-domains employing the commercially available Kubios software [10]. The selection of the Kubios filter has been usually performed subjectively. While some studies have selected and reported the use of a Low or Medium Kubios filters for the same cohort, others did not report the filter employed. Based on the results of the current study, it should be mandatory to explicitly acknowledge which Kubios filter is used in order to ease comparability of HRV derived parameters among different studies.

To the best of our knowledge, there is only one study testing the impact of the application of different Kubios filters on the quantification of the HRV derived parameters obtained by short-term recordings [13]. Aranda et al. [13] found that the use of a Very Strong filter (compared to the others filters) significantly affected the quantification of HRV derived parameters in time- and frequency-domains in 30 professional athletes. These findings concur with ours, and in fact we observed a significant impact on HRV derived parameters in time- and frequency-domains among the Kubios filters even using the Very Low, the Low, the Medium and the Strong filters (see Figure 2 and Figure 3). Despite the participants of the Aranda et al. study [13] were young adults (25 ± 3 years old), the different results obtained between studies could be explained by the training status (professional athletes vs. sedentary), methodological factors such as the body positioning during the HRV assessment (sitting vs. lying), or the heart rate monitor employed for the HRV recordings (Firstbeat Bodyguard vs. Polar RS800CX).

The clinical relevance of the observed differences on the HRV derived parameters should be considered. SDNN in resting conditions is considered a good indicator of cardiovascular and mortality risk [1]. Indeed, a previous study has reported that each increase of 10 ms in SDNN could indicate a reduction of ≈20% in the risk of mortality in adults with ischemic cardiomyopathy [30]. Comparing the SDNN values after the application of two similar Kubios filters in our study (the Very Low filter vs. the Low filter), we observed a mean SDNN difference of 3.66 ms, 1.16 ms and 0.08 ms in children, young and middle-aged adults. Therefore, these differences between filters might not have a clinically relevant impact. However, when extreme Kubios filters were compared (i.e., the Very Low filter vs. the Very Strong filter), we observed a mean SDNN difference of 38.68 ms, 27.25 ms and 7.83 ms in children, young and middle-aged adults. These mean SDNN differences between filters likely represent a clinical relevancy difference on both children and young adults, considering the aforementioned 10 ms in SDNN suggesting a reduction on the risk of mortality. Of note, the clinical relevancy mentioned should be considered with caution as some important differences need to be acknowledged: the cohorts of the study (adults with cardiovascular diseases vs. children, young and middle-aged adults without cardiovascular diseases), the length and instrument employed during the HRV assessment (24 h Holter vs. 10–15 min Polar RS800CX heart rate monitor). Furthermore, in practice, could be inappropriate to compare SDNN values because the total variance of HRV increases with the duration of the HRV recording [1].

The Kubios user’s guide recommends a percentage of interpolated R-R intervals lower than 5%. The software developers argue that this percentage should be enough to remove artefacts avoiding an excessive interpolation of R-R intervals. To our knowledge, this recommendation has not been tested in large and/or different cohorts. Moreover, they suggested that the Kubios filter should be individually selected to account for inter-individual variability in the HRV. Based on our results, we suggested the use a lower intensity Kubios filter (i.e. Very Low, Low or Medium filters) in younger populations, while a stronger Kubios filter could be used in older populations (with caution when using the Very Strong Kubios filter) to achieve the manufacturer’ recommendation of less than 5% of R-R intervals interpolated to remove HRV recordings artefacts (see Table 2). We also observed that the Very Strong filter highly reduces the HRV, because by definition, it must exclude every R-R interval which is 50ms longer or shorter than the local average R-R interval. Thus, the Very Strong filter equalizes the HRV among the study participants (Figure S4, Figure S5 and Figure S6).

The design of the current study precludes providing definitive thresholds and future studies are needed testing those recommendations. Moreover, we do not know whether Kubios filters remove “real” artefacts or large prolongations of R-R interval which may appear in healthy young individuals. Therefore, for Kubios software users, it is important to choose a reasonable filtering level (e.g., with caution when using the Very Strong Kubios filter), and hence, this study may have practical implications. Indeed, the kind of filter should correspond to the quality of the data, e.g., data collected during exercises or daily activity will need stronger filters than those recorded at rest. In addition, according to the results of our study, one should also consider the age of subjects before choosing the appropriate filter. Furthermore, we would recommend to the Kubios software users to apply different filters and to perform sensitivity analyses in order to study the effect of a treatment “*X*” on the HRV derived parameters. Thus, the Kubios software users might have a higher confident level in their results because are not “filter dependent” (i.e., their results are consistent across different Kubios filters).

We are aware of some limitations in our study: (1) the children cohort included in the present study was a sample of sedentary children with overweight/obesity, so we cannot extrapolate our findings to normal-weight children, (2) the experimental conditions during the HRV assessment were not exactly the same in the three different studies (e.g., the hour and/or dates of the HRV assessments), (3) the HRV assessment was performed once per participant during a relatively short period (10–15 min), and (4) we did not employ ECG tracings to verify whether “real” artefacts happened or not, thus our results could be influenced by the fact that we have used a polar RS800CX. Therefore, future studies should be performed using other alternatives tools (e.g., a different heart rate monitor or ECG) to confirm whether the impact of the different Kubios filters remained independently of the tool employed for recording the heart rhythm. On the other hand, the strengths of our study were: (1) a large sample size; (2) the impact of different Kubios filters was tested in three different and independent human cohorts; (3) all R-R interval signals have been visually inspected to detect obvious artefacts; and, (4) the HRV derived parameters were obtained from short-term recordings, which are increasing their popularity in both clinical and research context due their relative ease in data collection and management.

## 5. Conclusions

The results of the present study suggest that the application of different Kubios filters had a significant impact on the quantification of HRV derived parameters obtained from short-term recordings in both time and frequency-domains in children, young and middle-aged adults. Moreover, reporting the filter employed should be mandatory to allow comparisons across studies. Although the design of the current study precludes providing definitive thresholds, we suggested to use a Very Low, Low or Medium filter in children and young adults, whereas any Kubios filter (with caution when using the Very Strong Kubios filter) may be employed for middle-aged adults, when the processing of the short-terms HRV recordings is performed using the commercially available Kubios software. Further studies are guaranteed in order to elucidate which are the best Kubios filters to obtain valid data of HRV derived parameters.

## Figures and Tables

**Table 1:** Descriptive data of the participants.

	**Children (*n* = 107)**			**Young Adults (*n* = 132)**			**Middle-Aged Adults (*n* = 73)**		
	*n*		%	*n*		%	*n*		%
Sex									
Male	60		57.1	43		32.6	35		47.9
Female	45		42.9	89		67.4	38		52.1
	Mean	±	SD	Mean	±	SD	Mean	±	SD
Age (years)	10	±	1	22	±	2	54	±	5
Body mass index (kg/m^2^)	26.9	±	3.7	25.0	±	4.8	26.7	±	3.8
Waist circumference (cm)	90.2	±	9.9	81.6	±	14.6	95.0	±	11.8

Data are presented as mean and standard deviation (SD) unless otherwise stated.

**Table 2:** Percentage of R-R intervals interpolated in the heart rate variability (HRV) measurements when using different Kubios filter.

	**Children (*n* = 107)**					**Young Adults (*n* = 132)**					**Middle-Aged Adults** **(*n* = 73)**				
	**Mean**	**±**	**SD**	**Min**	**Max**	**Mean**	**±**	**SD**	**Min**	**Max**	**Mean**	**±**	**SD**	**Min**	**Max**
Beats corrected (%)															
During the whole measurement (10–15 min)															
None	0.0	±	0.0	0.0	0.0	0.0	±	0.0	0.0	0.0	0.0	±	0.0	0.0	0.0
Very Low	0.8	±	1.7	0.0	11.8	0.2	±	0.7	0.0	5.3	0.1	±	0.1	0.0	0.8
Low	1.6	±	2.6	0.0	15.5	0.4	±	1.1	0.0	5.4	0.1	±	0.2	0.0	1.0
Medium	3.4	±	4.4	0.0	20.9	1.3	±	2.7	0.0	15.6	0.1	±	0.4	0.0	2.8
Strong	9.0	±	9.2	0.0	36.9	5.2	±	6.9	0.0	35.4	0.8	±	1.7	0.0	9.3
Very Strong	41.0	±	18.1	1.9	76.6	35.0	±	17.2	1.1	74.8	14.9	±	11.5	0.06	45.7
During the selected period (5 min)															
None	0.0	±	0.0	0.0	0.0	0.0	±	0.0	0.0	0.0	0.0	±	0.0	0.0	0.0
Very Low	0.7	±	1.8	0.0	11.6	0.1	±	0.7	0.0	7.7	0.1	±	0.3	0.0	1.5
Low	1.5	±	3.0	0.0	17.9	0.4	±	1.1	0.0	7.7	0.2	±	0.8	0.0	5.2
Medium	3.2	±	5.0	0.0	25.3	1.2	±	2.7	0.0	17.1	0.4	±	1.5	0.0	8.8
Strong	8.5	±	9.7	0.0	39.6	4.5	±	7.1	0.0	36.1	1.7	±	4.4	0.0	21.0
Very Strong	39.4	±	19.3	1.0	77.2	33.1	±	18.8	0.0	75.5	17.1	±	15.8	0.0	60.8

Data are presented as mean and standard deviation (SD), minimum (Min) and maximum (Max).

## Supplementary Materials

The following are available online at https://www.mdpi.com/2077-0383/9/2/325/s1, Figure S1: Example of a visual inspection of a R-R signal to find possible artefacts or premature contractions across Kubios filters; Figures S2 and S3: Differences on the Heart Rate Variability (HRV) time- and frequency-domains parameters respectively without considering the Very Strong filter; Figure S4: Differences between cohorts on the SDNN using different Kubios filters; Figure S5: Differences between cohorts on the pNN50 using different Kubios filters; and Figure S6: Differences between cohorts on the HF using different Kubios filters.
