# Supplementary material for: Impact of Using Different Levels of Threshold-Based Artefact Correction on the Quantification of Heart Rate Variability in Three Independent Human Cohorts
Source: J Clin Med. 2020 Jan 23;9(2):325. doi: 10.3390/jcm9020325 (PMC7074236; doi:10.3390/jcm9020325)
Supplement: Supplementary file 1 [file jcm-09-00325-s001.pdf]

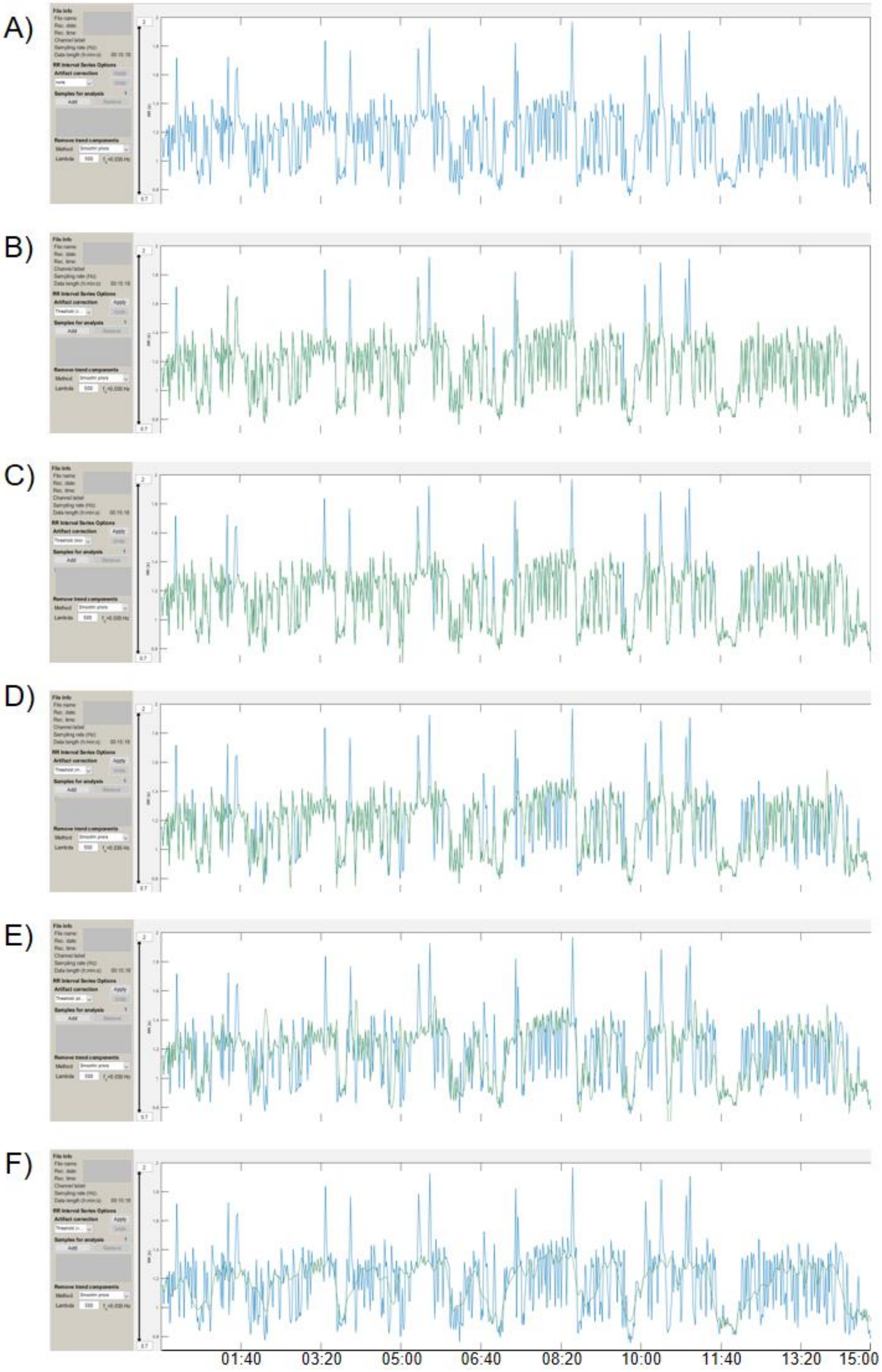

Figure S1. Example of a visual inspection of a R-R signal to find possible artifacts or premature contractions across Kubios filters (Panel A: None Filter; Panel B: Very Low Filter; Panel C: Low

Filter; Panel D: Medium Filter; Panel E: Strong Filter; and Panel F: Very Strong Filter). Green line represents the R-R signal before using a Kubios filter. Blue line (except when using the None Filter; Panel A) represent the excluded R-R signal after using the specific Kubios Filter.

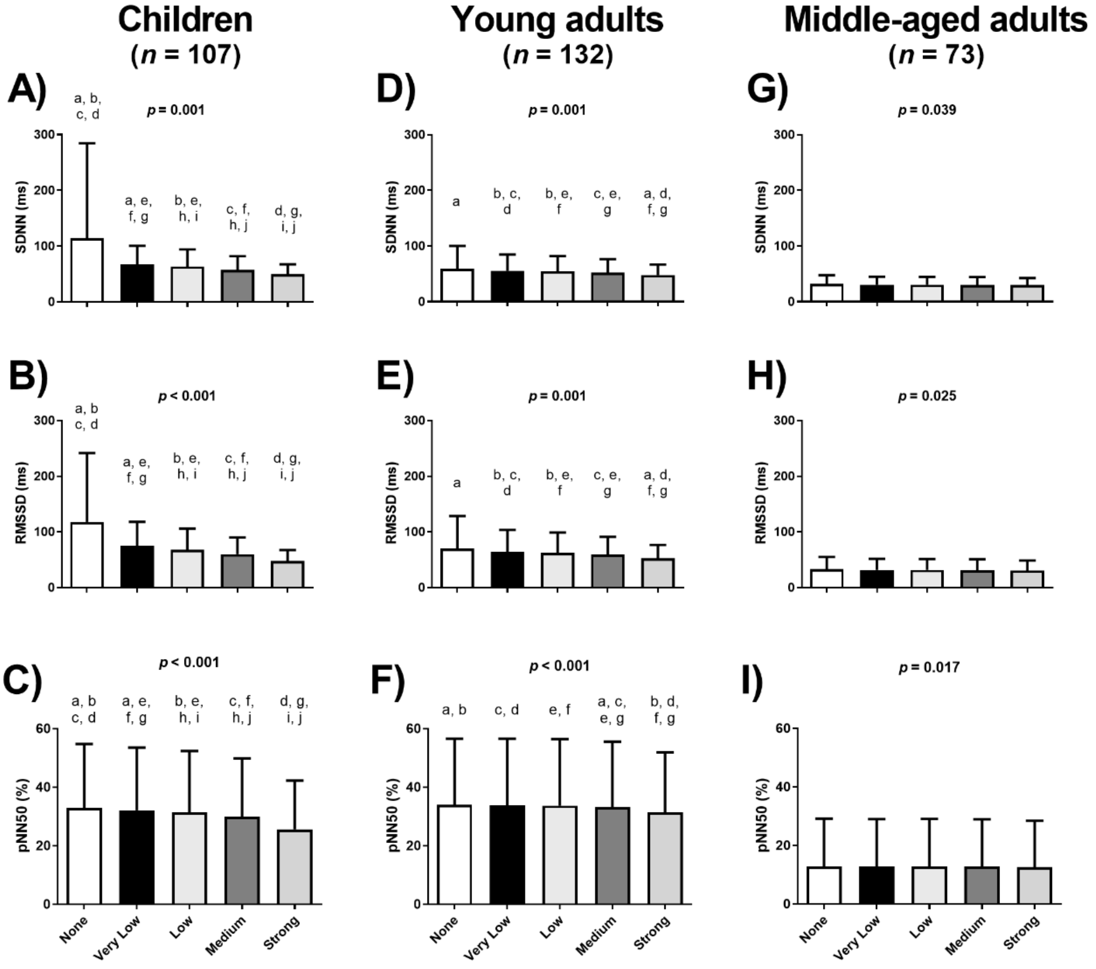

Figure S2. Differences on the Heart Rate Variability (HRV) time-domain parameters using different Kubios filters (excluding the Very Strong filter) in three different cohorts. Data are represented as mean and standard deviation. SDNN: standard deviation of all normal R–R intervals (Panels A, D and G); RMSSD: squared root of the mean of the sum of the squares of successive normal R–R interval differences (Panels B, E and H); pNN50: number of pairs of adjacent normal R–R intervals differing by more than 50ms in the entire recording (Panels C, F and I); *p* value from the ANOVA comparisons; similar letters means Bonferroni post-hoc differences.

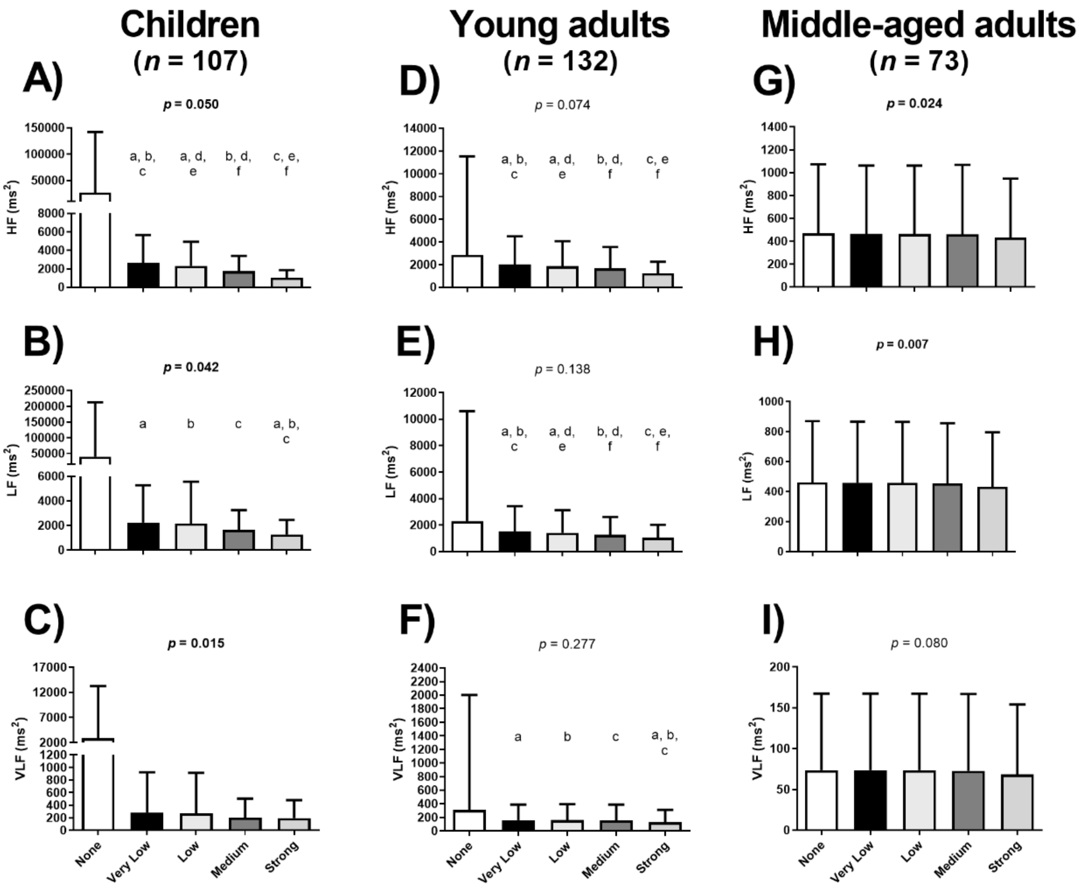

Figure S3. Differences on the Heart Rate Variability (HRV) frequency-domain parameters using different Kubios filter (excluding the Very Strong filter) in three different cohorts. Data are represented as mean and standard deviation. HF: power in the high frequency (in absolute units, ms^2; Panels A, E, and I); LF: power in the low frequency (in absolute units, ms^2; Panels B, F, and J); LF/HF: ratio of the power in the low frequency divided by the power in the high frequency (Panels C, G, and K); VLF: power in the very low frequency (in absolute units, ms^2; Panels D, H, and L). *p* value from the ANOVA comparisons; similar letters means Bonferroni post-hoc differences.

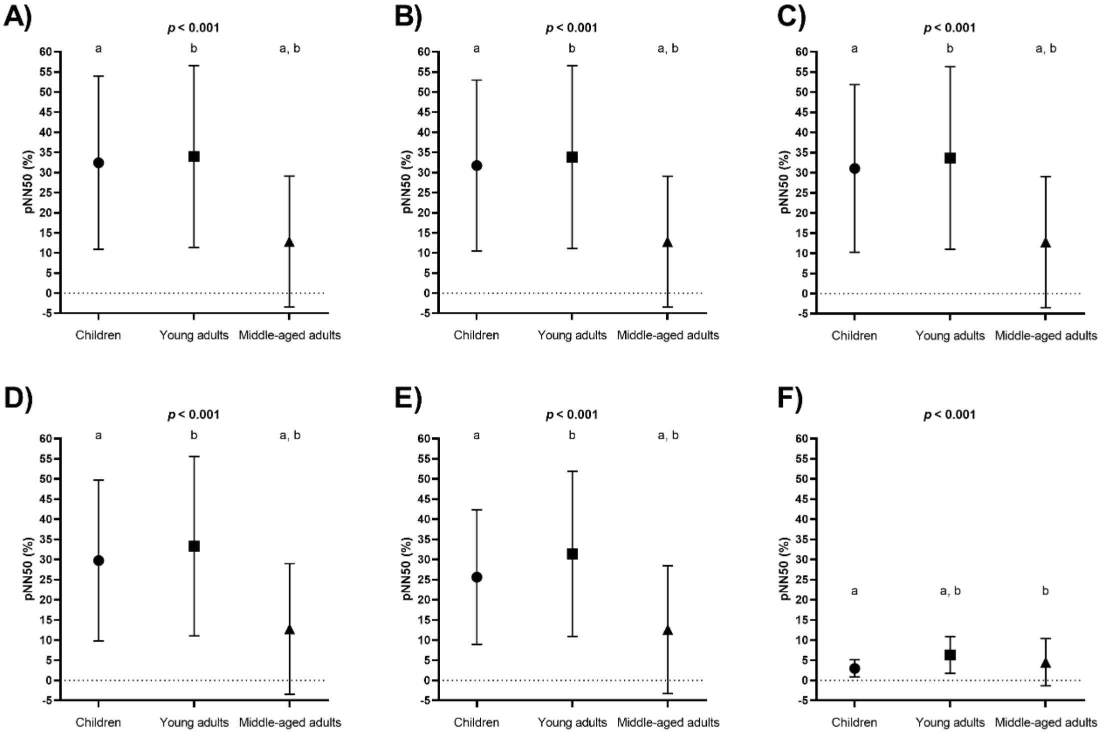

Figure S4. Differences between cohorts on the SDNN (standard deviation of all normal R–R intervals) using different Kubios filters (Panel A: None Filter; Panel B: Very Low Filter; Panel C: Low Filter; Panel D: Medium Filter; Panel E: Strong Filter; and Panel F: Very Strong Filter). Data are represented as mean and standard deviation of non-normalized SDNN; the results of SDNN after logarithmic normalization were similar. *p* value from the ANOVA comparisons; similar letters means Bonferroni *post-hoc* differences.

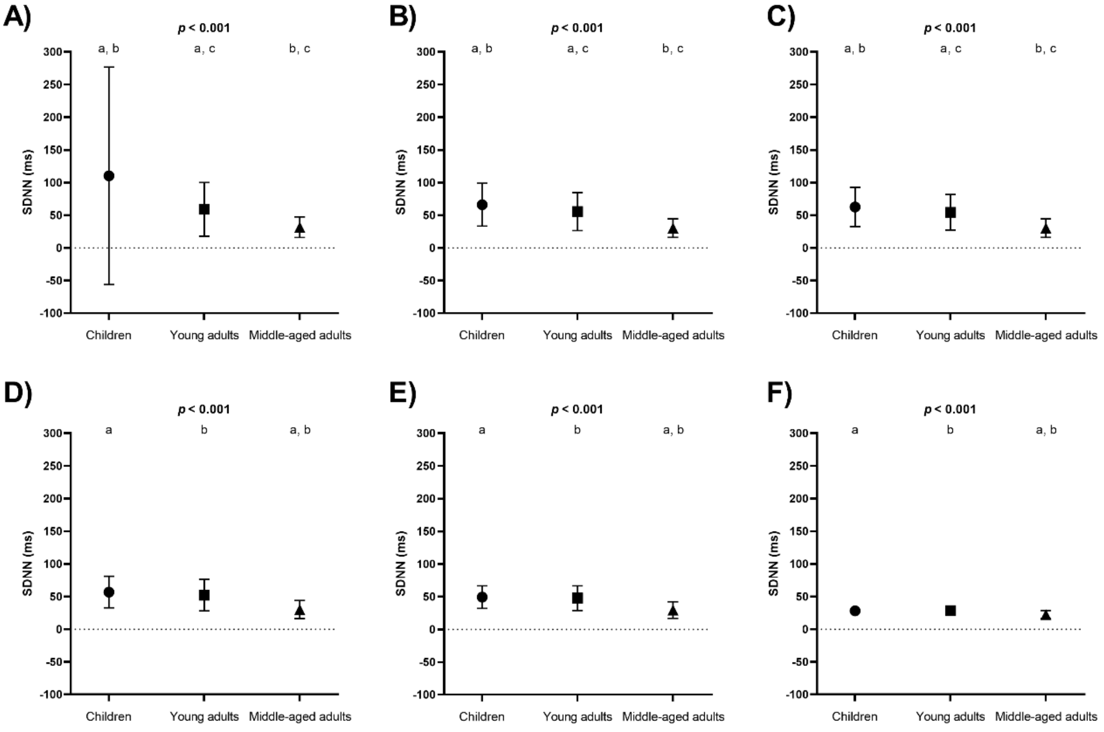

Figure S5. Differences between cohorts on the pNN50 (number of pairs of adjacent normal R–R intervals differing by more than 50ms in the entire recording) using different Kubios filters (Panel A: None Filter; Panel B: Very Low Filter; Panel C: Low Filter; Panel D: Medium Filter; Panel E: Strong Filter; and Panel F: Very Strong Filter). Data are represented as mean and standard deviation of non-normalized pNN50; the results of pNN50 after logarithmic normalization were similar. *p* value from the ANOVA comparisons; similar letters means Bonferroni *post-hoc* differences.

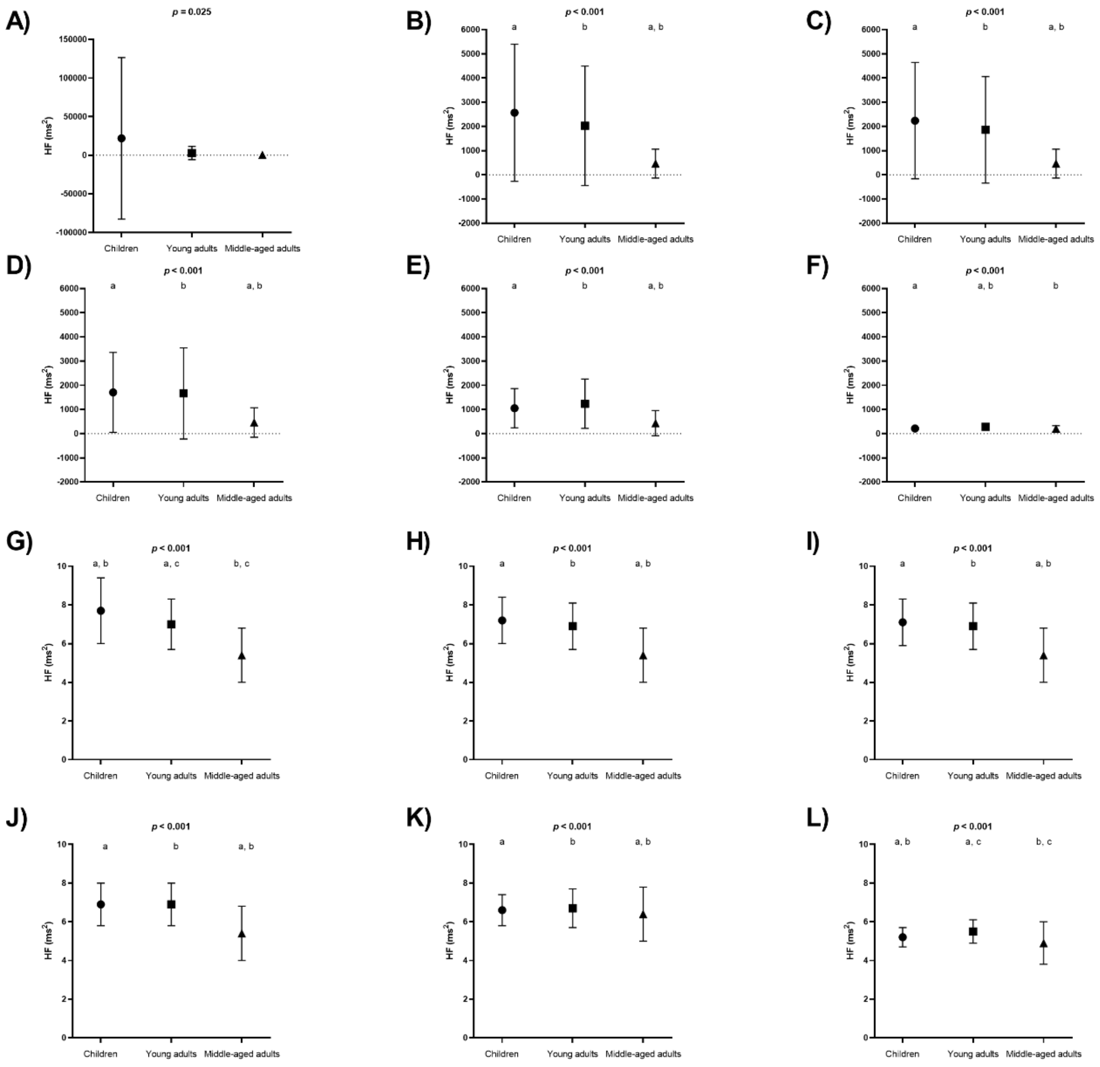

Figure S6. Differences between cohorts on the HF: power in the high frequency (in absolute units, ms^2) using different Kubios filters. Data are represented as mean and standard deviation of non-normalized HF (Panels A to F; Panel A: None Filter; Panel B: Very Low Filter; Panel C: Low Filter; Panel D: Medium Filter; Panel E: Strong Filter; and Panel F: Very Strong Filter); and after logarithmic normalization (Panels G to H; Panel G: None Filter; Panel H: Very Low Filter; Panel I: Low Filter; Panel J: Medium Filter; Panel K: Strong Filter; and Panel L: Very Strong Filter). *p* value from the ANOVA comparisons; similar letters means Bonferroni post-hoc differences.
